# Dehydroleucodine Induces ROS-Mediated Mitochondrial Apoptosis and G2/M Cell Cycle Arrest in Oral Squamous Cell Carcinoma

**DOI:** 10.7150/jca.127924

**Published:** 2026-06-04

**Authors:** Johnathon Lee, Tsung-Ming Chang, Chia-Jung Lee, Ju-Fang Liu, Hsin-Hua Chou

**Affiliations:** 1 School of Dentistry, College of Oral Medicine, Taipei Medical University, Taipei 110, Taiwan.; 2 School of Dental Technology, College of Oral Medicine, Taipei Medical University, Taipei City 110, Taiwan.; 3 Department of Otolaryngology Head and Neck Surgery, Shin-Kong Wu-Ho-Su Memorial Hospital, Taipei City 111, Taiwan.; 4 School of Oral Hygiene, College of Oral Medicine, Taipei Medical University, Taipei City 110, Taiwan.; 5 Translational Medicine Center, Shin-Kong Wu Ho-Su Memorial Hospital, Taipei City 111, Taiwan.; 6 Department of Medical Research, China Medical University Hospital, China Medical University, Taichung City 404, Taiwan.; 7 Department of Dentistry, Taipei Medical University Hospital, Taipei, 110, Taiwan.

**Keywords:** dehydroleucodine, oral squamous cell carcinoma, reactive oxygen species, mitochondrial apoptosis, G2/M cell cycle arrest, natural compounds

## Abstract

Oral squamous cell carcinoma (OSCC) is a highly aggressive malignancy with poor clinical outcomes due largely to recurrence and therapy resistance. Dehydroleucodine (DhL) is a sesquiterpene lactone derived from *Asteraceae* species, which has demonstrated anticancer activity in several tumor models. However, its therapeutic relevance in OSCC has not been established. This study investigated the antitumor effects of DhL and the mechanisms underlying its activity in OSCC using complementary *in vitro* and *in vivo* approaches. DhL significantly suppressed cell viability and clonogenic growth in HSC3 and SCC4 cells, with relatively low cytotoxicity toward normal gingival fibroblasts (HGF-1) under the tested conditions. The underlying mechanisms involved apoptotic cell death and G2/M phase arrest, accompanied by reduced cyclin B1 and CDK1 expression. DhL was also shown to promote mitochondrial dysfunction, as indicated by loss of mitochondrial membrane potential, increased Bax/Bcl-2 ratio, elevated cleaved caspase-3 expression and activity, and increased intracellular reactive oxygen species (ROS) generation. Pretreatment with the antioxidant N-acetylcysteine partially attenuated apoptosis and cell cycle arrest, suggesting that ROS plays a role in both responses. Systemic DhL treatment in an *in vivo* xenograft model significantly inhibited tumor growth and increased cleaved caspase-3 expression in tumor tissue without a significant loss of body weight. Together, these findings identify DhL as a potent suppressor of OSCC growth, which acts through ROS-associated mitochondrial apoptosis and G2/M arrest, supporting further preclinical evaluation of DhL as a candidate therapeutic agent for OSCC.

## Introduction

As the most common form of oral cancer, oral squamous cell carcinoma (OSCC), remains a critical public health challenge 1 with a global health burden of 389,000 new cases and 188,000 deaths annually 2. Despite advances in therapeutic strategies, the five-year survival rate for OSCC patients remains unsatisfactorily low 3, due in part to the limitations of current surgical methods, radiotherapy, and chemotherapy. These shortcomings frequently lead to recurrence, treatment resistance, and severe adverse effects 4-7. There is a particular need for new therapeutic agents for OSCC.

Natural compounds have long been the primary source of anticancer agents 8. Plants of the *Asteraceae* family have been extensively utilized in traditional medicine, and recent investigations have verified their diverse medicinal properties 9-12. Among the bioactive metabolites isolated from *Asteraceae* species, sesquiterpene lactones have garnered particular attention for their anti-inflammatory, antimicrobial, immunomodulatory, and anticancer activities 13-15. Dehydroleucodine (DhL) is a sesquiterpene lactone that has demonstrated anticancer activity against a number of malignancies. It has been shown to regulate multiple cellular processes associated with cancer cell survival, including apoptosis, cell cycle progression, and redox homeostasis 16-21.

Although DhL has demonstrated anticancer activity against other malignancies, its effects on OSCC and the underlying mechanisms remain poorly understood. Given the limited treatment options and poor outcomes in OSCC, together with strong preclinical evidence of DhL's anticancer activity, further investigation of DhL in OSCC is warranted. This study conducted a comprehensive series of *in vitro* assays to assess the effects of DhL on OSCC cell viability, apoptosis induction, cell cycle regulation, and oxidative stress modulation. A xenograft mouse model was also employed to assess its antitumor activity *in vivo*. These experiments were designed to characterize the anticancer effects of DhL in OSCC and to clarify the molecular mechanisms underlying its activity, thereby supporting further evaluation as a potential therapeutic agent for oral cancer.

## Materials and Methods

### Reagents and Chemicals

Dehydroleucodine (DhL, catalog no. D4196) and Calcein-AM (catalog no. 206700) were purchased from Sigma-Aldrich (St. Louis, MO, USA). DhL was dissolved in dimethyl sulfoxide (DMSO) to prepare a stock solution. For *in vitro* experiments, the stock solution was freshly diluted in culture medium to a specific working concentration immediately prior to use, with the same final concentration used across all groups. N-acetylcysteine (NAC), 4',6-diamidino-2-phenylindole (DAPI), and other chemicals were purchased from Sigma-Aldrich. MitoProbe^TM^ JC-1 Assay Kit, Fluo-3-pentaacetoxymethyl ester (Fluo-3 AM), and 2',7'-dichlorodihydrofluorescein diacetate (H_2_DCFDA) were purchased from Thermo Fisher Scientific (Waltham, MA, USA).

Primary antibodies against Bax (GTX100063), Bak (GTX109683), Bcl-2 (GTX100064), Bcl-xL (GTX105661), and β-actin were purchased from GeneTex (Hsinchu, Taiwan). Antibodies against cyclin B1 and CDK1 were obtained from Merck Millipore. Cleaved caspase-3 antibodies were purchased from Cell Signaling Technology (Danvers, MA, USA), and GAPDH antibodies were obtained from Proteintech (Wuhan, China). Horseradish peroxidase (HRP)-conjugated secondary antibodies against rabbit and mouse IgG were purchased from Merck Millipore. All other chemicals and reagents were purchased from Sigma-Aldrich (St. Louis, MO, USA) unless otherwise specified.

### Cell Culture

Human oral squamous cell carcinoma cell lines HSC3 (Merck KGaA, Darmstadt, Germany) and SCC4 (Bioresource Collection and Research Center, Hsinchu, Taiwan) as well as the normal human gingival fibroblast cell line HGF-1 (ATCC, Manassas, VA, USA) were cultured in high-glucose Dulbecco's Modified Eagle Medium (DMEM) supplemented with 10% fetal bovine serum (FBS; Thermo Fisher Scientific, Waltham, MA, USA) and 1% penicillin-streptomycin solution (100 U/mL penicillin and 100 μg/mL streptomycin; Thermo Fisher Scientific). Cells were maintained at 37 °C in a humidified atmosphere under 5% CO₂. Culture medium was refreshed at intervals of 48 h, and cells were subcultured at 70-80% confluence.

### Cell Viability Assay

Cell viability was assessed using the Cell Counting Kit-8 (CCK-8; Abbkine, KTA1020, Wuhan, China) in accordance with the manufacturer's protocol. HSC3, SCC4, and HGF-1 cells were seeded in 48-well plates (1 × 10⁴ cells/well) and treated with DhL (0, 2.5, 5, 10, and 20 μM) for 24 h. Following treatment, 10 μL of CCK-8 solution was added to each well and incubated at 37 °C for 4 h. Absorbance at 450 nm was recorded using a microplate reader (Varioskan™ LUX, Thermo Fisher Scientific, Waltham, MA, USA).

### Colony Formation Assay

Long-term clonogenic survival was assessed by seeding HSC3 and SCC4 cells in 6-well plates at a density of 500 cells per well and allowing them to adhere overnight. Cells were then treated with DhL (0, 2.5, 5, 10, or 20 μM) and refreshed with fresh drug-containing solution at intervals of 3 days. After culturing for 10-14 days, colonies were fixed using 4% paraformaldehyde for 15 min at room temperature and then stained with 0.5% crystal violet solution for 30 min. After photographing the plates, the crystal violet dye was solubilized with 33% acetic acid. Absorbance at 570 nm was measured using a microplate reader (Varioskan™ LUX) to quantify colony formation.

### Cell Morphology and Fluorescent Staining

HSC3 and SCC4 cells were seeded in 6-well plates at a density of 5 × 10⁵ cells per well and treated with DhL (0, 2.5, 5, 10, or 20 μM) for 24 h. Following treatment, cells were co-stained with 0.5 μM Calcein-AM (live cell marker), 3 μg/mL propidium iodide (PI; dead cell marker), and 7.5 μg/mL Hoechst 33258 (nuclear marker) at 37 °C for 30 min in the dark. Stained cells were visualized using a fluorescence microscope (Eclipse Ti, Nikon, Tokyo, Japan). Live cells exhibited green fluorescence (Calcein-AM) and dead cells presented red nuclear fluorescence (PI), while nuclear morphology was assessed via blue Hoechst staining.

### Apoptosis Assay

Apoptotic cell death was quantified by flow cytometry using Annexin V-FITC/PI dual staining. Briefly, HSC3 and SCC4 cells were seeded in 6-well plates (5 × 10⁵ cells/well) and treated with DhL (0, 2.5, 5, 10, or 20 μM) for 24 h. Cells were harvested via trypsinization, washed with phosphate-buffered saline (PBS), and resuspended in binding buffer. Cells were then stained with 5 μL Annexin V-FITC and 5 μL propidium iodide (Annexin V-FITC/PI Apoptosis Kit; Elabscience, E-CK-A211, Wuhan, China) and incubated for 15 min at room temperature in the dark in accordance with the manufacturer's instructions. Samples were analyzed by flow cytometry (Accuri C5, BD Biosciences, East Rutherford, NJ, USA). Early apoptotic cells were identified as Annexin V⁺/PI⁻, while late apoptotic/necrotic cells were identified as Annexin V⁺/PI⁺.

### Terminal deoxynucleotidyl transferase dUTP nick end labeling (TUNEL) Assay

DNA fragmentation, a hallmark of apoptosis, was assessed using the One-Step TUNEL Apoptosis Assay Kit (Green Fluorescence; Abbkine, KTA2010, Wuhan, China) in accordance with the manufacturer's protocol. Briefly, HSC3 and SCC4 cells were seeded in 6-well plates (5 × 10⁵ cells/well) and treated with DhL (0, 2.5, 5, 10, or 20 μM) for 24 hours. Cells were collected, fixed with 4% paraformaldehyde on ice for 30 min, and then permeabilized with 0.3% Triton X-100 for 5 min at room temperature. After washing with PBS, the cells were incubated with terminal deoxynucleotidyl transferase (TdT) labeling reaction mixture at 37 °C for 2 h in the dark. FITC-positive cells (indicating DNA fragmentation) were quantified via flow cytometry (Accuri C5, BD Biosciences, East Rutherford, NJ, USA).

### Cell Cycle Analysis

Cell cycle distribution was evaluated using propidium iodide (PI) staining and flow cytometry. Briefly, HSC3 and SCC4 cells were seeded in 6-well plates (5 × 10⁵ cells/well) and treated with DhL (0, 2.5, 5, 10, or 20 μM) for 24 h. Cells were harvested, washed with PBS, and fixed in ice-cold 70% ethanol at -20 °C for at least 2 h. Fixed cells were washed twice with PBS and resuspended in staining solution containing 200 μg/mL RNase A and 10 μg/mL propidium iodide (Sigma-Aldrich). After incubation at 37 °C for 30 min in the dark, cell cycle distribution was analyzed by flow cytometry (CytoFLEX, Beckman Coulter, Brea, CA, USA). The percentages of cells in G0/G1, S, G2/M, and sub-G1 (apoptotic) phases were quantified using the analysis software provided by the instrument manufacturer.

### JC-1 Mitochondrial Membrane Potential Assay

Mitochondrial membrane potential was assessed using the JC-1 fluorescent probe (Mitochondrial Membrane Potential Probe; Thermo Fisher Scientific, T3168). HSC3 and SCC4 cells were seeded in 6-well plates (5 × 10⁵ cells/well) and treated with DhL (0, 2.5, 5, 10, or 20 μM) for 24 h. Cells were then incubated with 5 μg/mL JC-1 dye at 37 °C for 30 min in the dark, washed twice with PBS, and immediately examined using a fluorescence microscope (Eclipse Ti, Nikon). In healthy cells with intact mitochondrial membrane potential, JC-1 forms aggregates that emit red fluorescence. Upon mitochondrial depolarization, JC-1 remains monomeric and emits green fluorescence. The red-to-green fluorescence ratio was used as an indicator of mitochondrial membrane integrity.

### Caspase-3 Activity Assay

Caspase-3 enzymatic activity was measured using the Caspase-3 Colorimetric Assay Kit (Abbkine, KTA3022, Wuhan, China). HSC3 and SCC4 cells (approximately 2 × 10⁶ cells per condition) were seeded in 10 cm dishes, grown to approximately 80% confluence, and treated with DhL (0, 2.5, 5, 10, or 20 μM) for 24 h. Following treatment, cells were harvested, washed with PBS, and lysed in Working Cell Lysis Buffer containing dithiothreitol (DTT) in accordance with the manufacturer's instructions. Lysates were incubated on ice for 30 min and centrifuged at 10,000 × *g* for 10 min at 4 °C. After determining protein concentrations in the supernatants, equal amounts of protein were transferred to a 96-well plate. Each sample was mixed with 50 μL of Working Reaction Buffer and 5 μL Ac-DEVD-pNA substrate (4 mM) and incubated at 37 °C for 2-4 h. Caspase-3 activity was quantified by measuring absorbance at 405 nm using a microplate reader (Varioskan™ LUX). Results were normalized to protein concentration and expressed as fold change relative to untreated controls.

### Intracellular ROS Detection

Intracellular ROS levels were assessed using the fluorescent probe CM-H₂DCFDA (General Oxidative Stress Indicator; Thermo Fisher Scientific, C6827). HSC3 and SCC4 cells were seeded in 6-well plates (4 × 10⁵ cells/well) and treated with DhL (0, 2.5, 5, 10, or 20 μM) for 24 h. Cells were then incubated with 0.5 μM CM-H₂DCFDA at 37 °C for 30 min in the dark. Cells were washed twice with PBS to remove excess probe residue. ROS-dependent fluorescence was assessed using fluorescence microscopy (Eclipse Ti, Nikon) and flow cytometry (Accuri C5). Increased green fluorescence intensity indicated elevated intracellular ROS levels.

To assess the contribution of oxidative stress to DhL-induced cellular effects, HSC3 and SCC4 cells were pretreated with the antioxidant N-acetylcysteine (NAC; 75 μM) for 1 h prior to treatment with DhL (20 μM). After combined NAC and DhL treatment for 24 h, cells were analyzed for viability (CCK-8 assay), apoptosis (Annexin V/PI staining), and cell cycle distribution (PI staining).

### Western Blotting

Cells were lysed in RIPA buffer (Thermo Fisher Scientific) containing protease and phosphatase inhibitor cocktails (Sigma-Aldrich). Protein concentrations were determined using the BCA Protein Assay Kit (Thermo Fisher Scientific). Equal quantities of protein (20-30 μg) were separated by sodium dodecyl sulfate-polyacrylamide gel electrophoresis (SDS-PAGE) on 10-12% gels and transferred to polyvinylidene difluoride (PVDF) membranes (Merck Millipore, Burlington, MA, USA). The membranes were blocked with 5% non-fat milk in Tris-buffered saline containing 0.1% Tween-20 (TBST) at room temperature for 1 h and then incubated with primary antibodies (1:1000 dilution) overnight at 4 °C. After washing with TBST, membranes were incubated with HRP-conjugated secondary antibodies (1:5000 dilution) for 1 h at room temperature. Immunoreactive bands were visualized using enhanced chemiluminescence (ECL) reagent and detected with a UVP chemiluminescence imaging system (Analytik Jena US, Upland, CA, USA). Band intensities were quantified using ImageJ software (NIH, Bethesda, MD, USA), and protein expression levels were normalized to GAPDH or β-actin, as indicated in the corresponding figures.

### *In Vivo* Tumor Xenograft Model

Male NOD/SCID mice (7 weeks old, weighing 18-20 g) were purchased from Bio-LASCO Co., Ltd. (Taipei, Taiwan) and housed under specific-pathogen-free conditions following a 12:12 h light/dark cycle at 22 ± 2 °C under 50 ± 10% relative humidity. Upon arrival, mice were randomly allocated to cages while maintaining a comparable distribution of body sizes across cages. Food and water were provided *ad libitum*, and the animals were acclimated for one week prior to experimentation. All animal care and experimental procedures were conducted in accordance with institutional guidelines and were approved by the Institutional Animal Care and Use Committee (IACUC) of Shin Kong Wu Ho-Su Memorial Hospital (approval no. SKHACC01000207).

For tumor establishment, 2 × 10⁶ HSC3 cells in 100 µL were injected subcutaneously into the right dorsal flank of each mouse 22, 23. Tumors were allowed to grow for 7 days until their volume reached approximately 50 to 100 mm^3^. To reduce baseline differences in tumor burden, mice with similar tumor volumes were randomly assigned to one of two groups (n = 8 per group): a vehicle control group or a DhL treatment group receiving 20 mg/kg DhL by intraperitoneal injection once daily for 16 consecutive days 18. For *in vivo* administration, DhL was formulated in a vehicle consisting of 10% DMSO, 40% PEG300, 5% Tween 80, and 45% sterile saline. The DhL stock solution was first mixed with PEG300, followed by Tween 80, and then diluted with sterile saline immediately prior to administration. Tumor dimensions were measured at intervals of 3 days using digital calipers, and tumor volumes were calculated using the following formula: tumor volume = (length × width²)/2. Body weight was monitored throughout the study as a general indicator of treatment tolerability. At the experiment endpoint (day 23 post-tumor implantation), mice were euthanized by CO₂ inhalation followed by cervical dislocation. Tumors were immediately excised, photographed, and weighed. Tumor tissues were either snap-frozen in liquid nitrogen for subsequent protein analysis or fixed in 10% neutral-buffered formalin for histological and immunohistochemical examination.

### Immunohistochemistry

Formalin-fixed, paraffin-embedded tumor tissues were cut into 3 μm sections, deparaffinized in xylene, and then rehydrated through a graded ethanol series to distilled water. Antigen retrieval was achieved by heating the sections in 10 mM sodium citrate buffer (pH 6.0) at 95-100 °C for 20 minutes. Endogenous peroxidase activity was quenched using a peroxidase blocking reagent (Novolink Polymer Detection System; Leica Biosystems, IL, USA). Afterward, nonspecific protein binding was minimized by applying blocking solution, and the sections were incubated with primary antibodies against Ki-67 or cleaved caspase-3 (GeneTex, USA) overnight at 4 °C. The following day, slides were exposed to Novolink polymer secondary antibodies for 1 hour at room temperature. Immunoreactive signals were visualized using 3,3'-diaminobenzidine (DAB) chromogen and counterstained with hematoxylin. The stained slides were examined under a light microscope, and immunoreactivity was evaluated semi-quantitatively using an H-score obtained by multiplying the percentage of positively stained tumor cells (0-100%) by the staining intensity score (0 to 3), yielding a total score ranging from 0 to 300.

### Statistical Analysis

Statistical analysis was performed using GraphPad Prism 9.0 (GraphPad Software, San Diego, CA, USA). Data are presented as mean ± SD from at least three independent experiments. Comparisons between two groups were analyzed using Student's t-test. For experiments involving more than two groups, statistical significance was determined by one-way ANOVA followed by the appropriate post hoc multiple-comparisons test according to the experimental design. Dunnett's multiple comparisons test was used for comparisons of multiple treatment groups with a single control group, whereas Tukey's multiple comparisons test was used when all pairwise comparisons among groups were required. Body weight and tumor volume measurements collected over time were analyzed using two-way ANOVA followed by Sidak's multiple comparisons test. A p value < 0.05 was considered statistically significant.

## Results

### Dehydroleucodine Reduces OSCC Cell Viability and Clonogenicity

**Figure 1:**
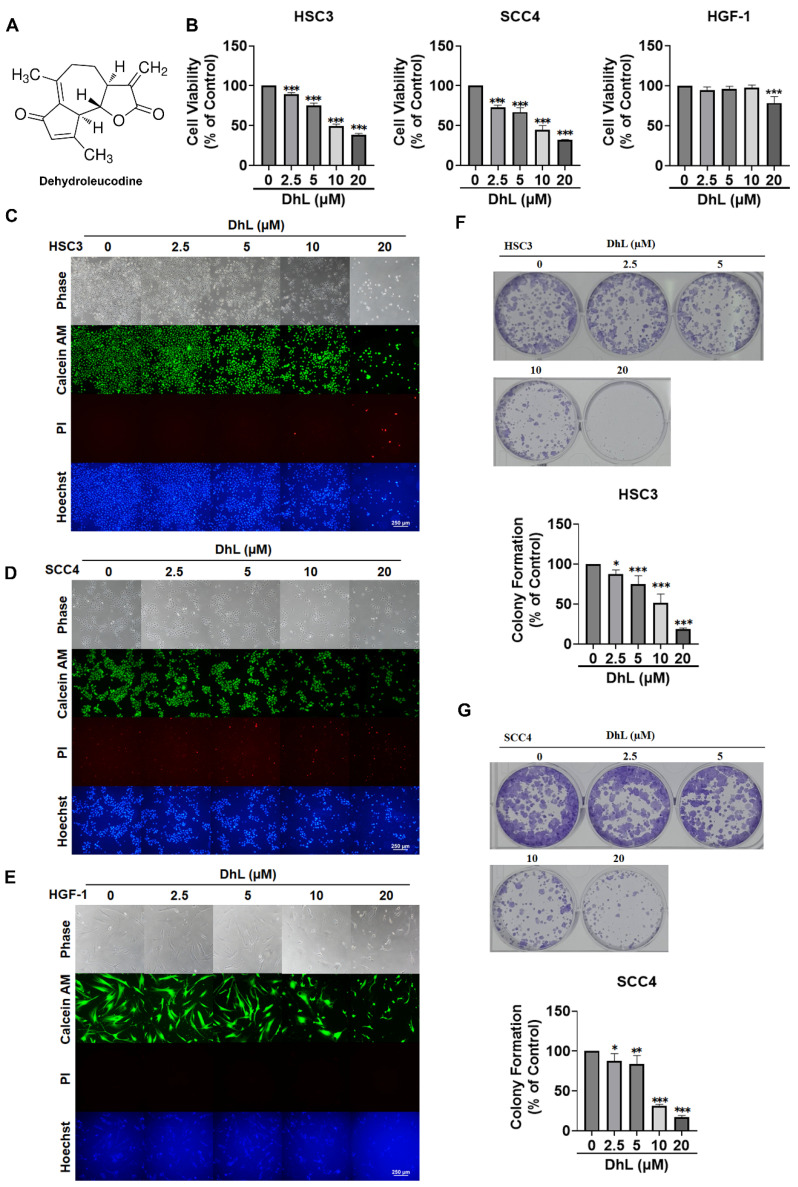
** DhL Reduces OSCC Cell Viability and Clonogenicity** (A) Chemical structure of DhL; (B) CCK-8 cell viability of HSC3, SCC4, and HGF-1 following treatment with increasing DhL concentrations (0, 2.5, 5, 10, 20 µM) over a period of 24 h; (C-E) Morphology and Calcein-AM/PI/Hoechst staining of HSC3 (C), SCC4 (D), and HGF-1 (E) after treatment at indicated DhL concentrations for 24 h. Scale bar: 250 µm; (F, G) Colony formation assays of HSC3 (F) and SCC4 (G) cells after DhL treatment for 10-14 days. Data are presented as mean ± SD from at least three independent experiments. Statistical significance was analyzed using one-way ANOVA followed by Dunnett's multiple comparisons test. *p < 0.05 vs. control, **p < 0.01 vs. control, and ***p < 0.001 vs. control.

The anticancer potential of DhL in OSCC was first examined by assessing its effects on cell viability using the CCK-8 assay. This involved treating HSC3 and SCC4 cells with increasing concentrations of DhL (0, 2.5, 5, 10, and 20 μM) over a period of 24 h. As shown in Figure 1A, DhL treatment induced a dose-dependent reduction in cell viability in both OSCC cell lines. At the highest concentration (20 μM), cell viability was reduced to 50-60% of control levels. DhL showed lower cytotoxicity toward normal human gingival fibroblasts (HGF-1) at the same concentrations (Figure 1B). Morphological examination revealed that DhL-induced cellular changes characteristic of cell death. Fluorescence microscopy revealed a dose-dependent decrease in the number of Calcein-AM-positive (viable) cells and a corresponding increase in the number of PI-positive (dead) cells (Figures 1C and 1D). Hoechst staining revealed condensed and fragmented nuclei, a hallmark of apoptotic cell death. These morphological alterations became increasingly pronounced with an increase in DhL concentration. Importantly, HGF-1 cells displayed limited morphological changes and PI staining under the tested conditions (Figure 1E).

The long-term impact of DhL on OSCC cell proliferation was assessed using colony formation assays. DhL treatment resulted in a dose-dependent suppression of clonogenic capacity in both HSC3 and SCC4 cells (Figures 1F and 1G). At concentrations of 10 μM and above, colony formation was nearly abolished, demonstrating that DhL impaired the long-term proliferative potential of OSCC cells. Collectively, these findings demonstrate that DhL inhibits OSCC cell growth with limited cytotoxicity toward HGF-1 cells under the tested conditions.

### Dehydroleucodine Induces Apoptosis and G2/M Cell Cycle Arrest in OSCC Cells

**Figure 2:**
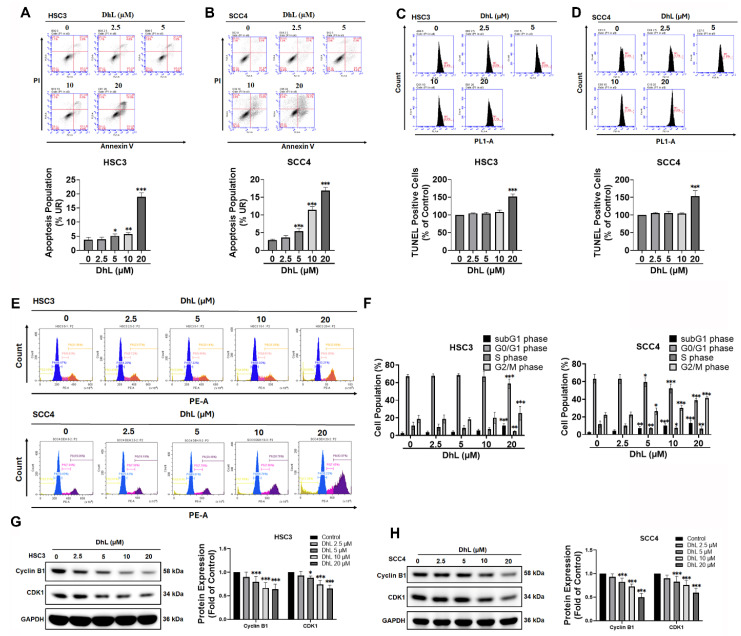
** DhL Induces Apoptosis and G2/M Cell Cycle Arrest in OSCC Cells** (A, B) Annexin V/PI staining of HSC3 (A) and SCC4 (B) cells after exposure to DhL for 24 h at indicated concentrations; (C, D) Flow cytometric analysis and quantification of TUNEL-positive HSC3 (C) and SCC4 (D) after DhL treatment for 24 h, selected on the basis of flow cytometry results; (E, F) Flow cytometry analysis of cell cycle distribution in HSC3 (E) and SCC4 (F) after exposure to DhL for 24 h; (G, H) Western blot analysis of cyclin B1 and CDK1 expression in HSC3 (G) and SCC4 (H) cells after DhL treatment for 24 h. Data are presented as mean ± SD from at least three independent experiments. Statistical significance was analyzed by one-way ANOVA followed by Dunnett's multiple comparisons test. *p < 0.05 vs. control, **p < 0.01 vs. control, and ***p < 0.001 vs. control.

Annexin V/PI dual staining was used to assess apoptosis in order to explore the mechanism underlying the DhL-induced reduction in cell viability. Flow cytometric analysis revealed a dose-dependent increase in the Annexin V⁺/PI⁺ population in both HSC3 and SCC4 cells, indicating late-stage apoptosis (Figures 2A and 2B). The relatively low proportion of early apoptotic cells (Annexin V⁺/PI⁻) across all treatment groups suggests that DhL predominantly induced late-stage apoptosis. These findings were supported by TUNEL assays, which detected DNA fragmentation associated with apoptotic cell death. DhL treatment significantly increased the percentage of TUNEL-positive cells in both cell lines, with the most pronounced effect at 20 μM (Figures 2C and 2D).

Considering the close association between apoptosis and cell cycle regulation, we examined the effect of DhL on cell cycle distribution. Propidium iodide staining and flow cytometry revealed a marked accumulation of cells in the G2/M phase in both HSC3 and SCC4 cells (Figures 2E and 2F). Concurrently, the proportion of cells in the G0/G1 phase decreased, while the sub-G1 population (representing apoptotic cells with fragmented DNA) increased in a dose-dependent manner. These observations indicate that DhL induced G2/M cell cycle arrest in addition to triggering apoptosis. To confirm G2/M cell cycle arrest, Western blot analysis was performed to examine the expression of key mitotic regulators, cyclin B1 and CDK1. As shown in Figures 2G and 2H, DhL treatment resulted in a dose-dependent downregulation of both cyclin B1 and CDK1 in HSC3 and SCC4 cells. These findings are consistent with G2/M arrest, indicating that DhL impairs mitotic progression through the downregulation of proteins essential for cell cycle regulation. Collectively, these results demonstrate that DhL reduces OSCC cell viability while inducing apoptosis and disrupting cell cycle progression at the G2/M checkpoint.

### DhL Disrupts Mitochondrial Membrane Potential and Activates the Intrinsic Apoptotic Pathway

**Figure 3:**
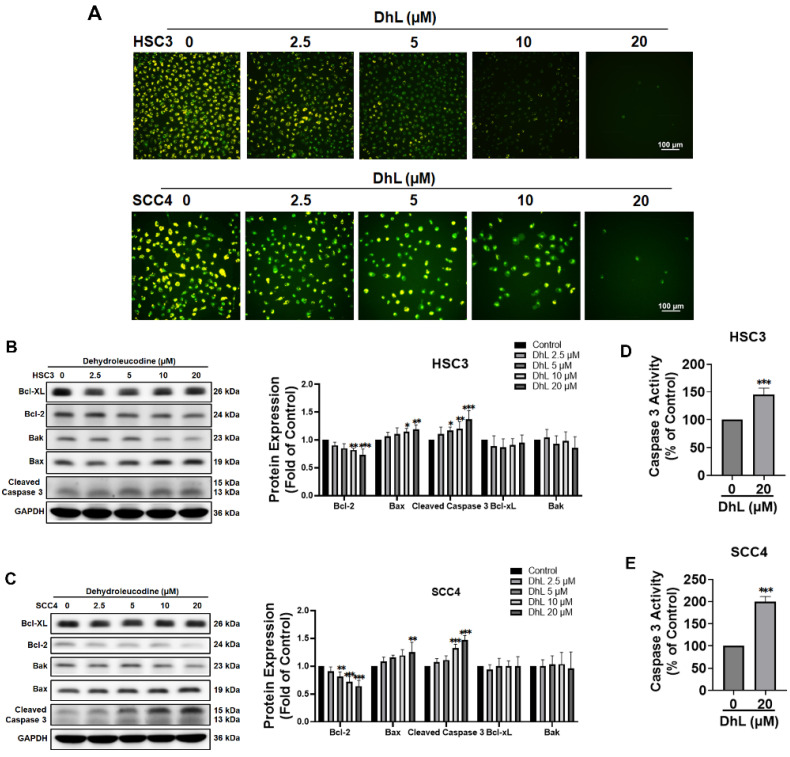
** DhL Disrupts Mitochondrial Integrity and Activates Apoptotic Pathways** (A) JC-1 assay for mitochondrial membrane potential in HSC3 and SCC4 cells after treatment with DhL for 24 h at indicated concentrations. Scale bar: 100 μm**;** (B, C) Western blot analysis of Bcl-xL, Bcl-2, Bax, Bak, and cleaved caspase-3 expression in HSC3 (B) and SCC4 (C) cells after treatment with DhL for 24 h. GAPDH was used as the loading control; (D, E) Caspase-3 activity assay in HSC3 and SCC4 cells after DhL treatment for 24 h. Data are presented as mean ± SD from at least three independent experiments. Statistical significance was analyzed by one-way ANOVA followed by Dunnett's multiple comparisons test. *p < 0.05 vs. control, **p < 0.01 vs. control, and ***p < 0.001 vs. control.

To determine whether DhL-induced apoptosis involves mitochondrial dysfunction, JC-1 staining was used to assess changes in mitochondrial membrane potential. In healthy cells, JC-1 forms aggregates in polarized mitochondria and emits red fluorescence, whereas in depolarized mitochondria, JC-1 remains monomeric and exhibits green fluorescence. As shown in Figure 3A, DhL treatment induced a dose-dependent shift from red to green fluorescence in both HSC3 and SCC4 cells, indicating progressive mitochondrial depolarization. This effect was evident at ≥ 5 μM and pronounced at 20 μM. The molecular mechanisms underlying DhL-induced mitochondrial apoptosis were examined by measuring the expression levels of key Bcl-2 family proteins that regulate permeabilization of the outer mitochondrial membrane. Western blot analysis revealed that DhL treatment significantly upregulated the pro-apoptotic protein Bax in a dose-dependent manner, while concurrently downregulating the anti-apoptotic protein Bcl-2 (Figures 3B and 3C). The resulting increase in Bax/Bcl-2 ratio is consistent with enhanced mitochondrial apoptotic signaling. Note that the expression levels of Bak and Bcl-xL were relatively unchanged, regardless of the treatment concentration. Consistent with activation of the mitochondrial apoptotic pathway, DhL treatment markedly increased cleaved caspase-3 expression and significantly enhanced caspase-3 enzymatic activity, particularly at 20 μM (Figures 3D and 3E). Collectively, these findings indicate that DhL-induced apoptosis in OSCC cells is associated with mitochondrial membrane depolarization, modulation of Bcl-2 family proteins, and caspase-3 activation.

### DhL-Induced Oxidative Stress Contributes to Apoptosis and G2/M Cell Cycle Arrest in OSCC Cells

**Figure 4:**
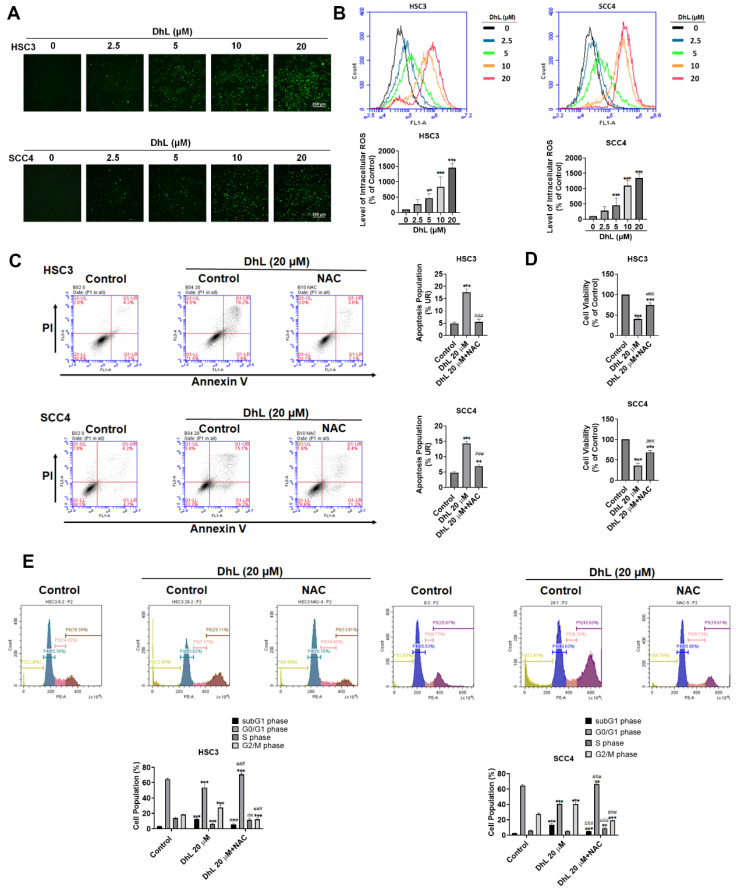
** DhL Induces Oxidative Stress in OSCC Cells** (A) CM-H₂DCFDA fluorescence microscope images of HSC3 and SCC4 cells after treatment with DhL for 24 h at indicated concentrations. Scale bar: 250 μm; (B) Flow cytometry analysis of ROS levels (CM-H₂DCFDA) in HSC3 and SCC4 cells after DhL exposure for 24 h; (C) Annexin V/propidium iodide (PI) staining of HSC3 and SCC4 cells after treatment with DhL for 24 h with or without 1 h NAC pretreatment, based on flow cytometry; (D) CCK-8 cell viability assay of HSC3 and SCC4 cells after treatment with DhL for 24 h, with or without 1 h NAC pretreatment; (E) PI staining and flow cytometry cell cycle analysis of HSC3 and SCC4 cells after treatment with DhL for 24 h, with or without 1 h NAC pretreatment. Data are presented as mean ± SD from at least three independent experiments. Statistical significance was analyzed by one-way ANOVA followed by Tukey's multiple comparisons test. *p < 0.05 vs. control, **p < 0.01 vs. control, and ***p < 0.001 vs. control; #*p* < 0.05 vs. DhL alone, ##*p* < 0.01 vs. DhL alone, and ###*p* < 0.001 vs. DhL alone.

Mitochondrial dysfunction is often associated with the elevated generation of reactive oxygen species (ROS) 24, 25. This study measured intracellular ROS levels using the fluorescence probe, H₂DCFDA, to determine whether DhL induces oxidative stress in OSCC cells. Fluorescence microscopy and flow cytometric analysis revealed that DhL treatment induced a dose-dependent ROS increase in both HSC3 and SCC4 cells, as indicated by the increased green fluorescence intensity in Figures 4A and 4B. Significant ROS accumulation was observed at 5 μM DhL and peaked at 20 μM. Quantitative flow cytometry confirmed that treatment with 20 μM DhL significantly increased the ROS-positive cell population relative to untreated controls. To determine whether ROS accumulation contributed to the effects of DhL, cells were pretreated with the antioxidant N-acetylcysteine (NAC) prior to DhL treatment. Annexin V/PI flow cytometry showed that NAC co-treatment markedly decreased the proportion of DhL-induced apoptotic cells (Figure 4C). NAC pretreatment significantly restored cell viability in DhL-treated cells, which suggests that oxidative stress is an important contributing factor to DhL-induced cytotoxicity (Figure 4D). Cell cycle analysis revealed that NAC pretreatment attenuated DhL-induced G2/M arrest and substantially reduced the sub-G1 apoptotic population (Figures 4E). These findings indicate that oxidative stress plays a key role in the pro-apoptotic and cell cycle-disruptive effects of DhL in OSCC cells, and that ROS scavenging can partially rescue cells from these effects.

### DhL Suppresses Tumor Growth and Induces Apoptotic Activity in an OSCC Xenograft Model

**Figure 5:**
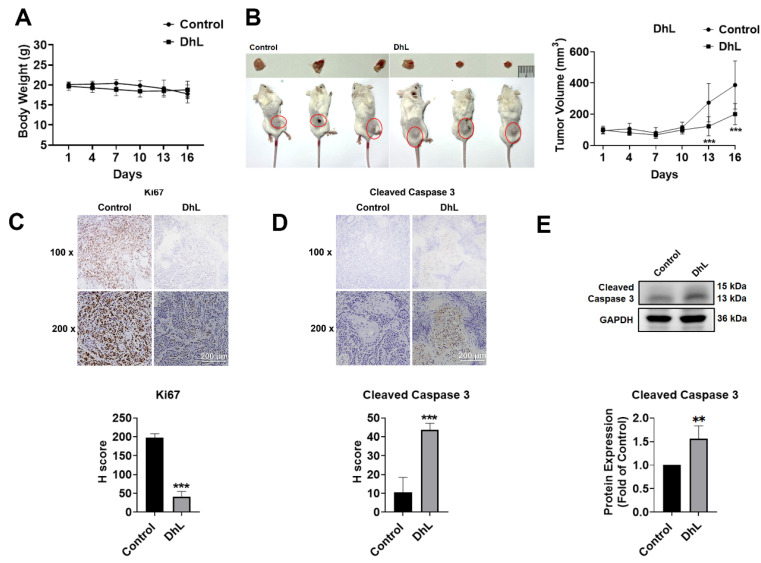
***In Vivo* Antitumor Effects of DhL in the OSCC Xenograft Model** (A) Changes in body weight in control and DhL-treated mice throughout the experimental period; (B) Representative image of excised tumors from each treatment group at the end of the experiment (left) and tumor volume over time (right), calculated as (length × width²) / 2; (C) IHC staining and H-score quantification of Ki-67 expression in tumors from DhL-treated mice and the control group (n=4). Scale bar: 200 μm; (D) IHC staining and H-score quantification of cleaved caspase-3 expression in tumors from DhL-treated mice and the control group (n=4). Scale bar: 200 μm; (E) Western blot analysis of cleaved caspase-3 expression in tumor tissues at study endpoint, with GAPDH used as loading control (n=4). Body weight and tumor volume were analyzed over time using two-way ANOVA followed by Sidak's multiple comparisons test. For endpoint comparisons, statistical significance was analyzed using Student's t-test. *p < 0.05 vs. control, **p < 0.01 vs. control, and ***p < 0.001 vs. control.

Our *in vitro* findings were corroborated by examining the *in vivo* antitumor activity of DhL in a NOD/SCID xenograft mouse model involving HSC3 cells. Beginning at 7 days after tumor implantation, mice were randomly assigned to receive either vehicle alone or DhL at 20 mg/kg by intraperitoneal injection once daily for 16 consecutive days. No significant between-group differences in body weight were observed throughout the treatment period, indicating that the DhL regimen was tolerated under the tested conditions (Figure 5A). As shown in Figure 5B, tumors harvested from DhL-treated mice were visibly smaller than those from control animals at the experiment endpoint. Quantitative analysis of tumor volumes revealed a significant decrease in tumor growth in the DhL-treated group, compared with the control group. Immunohistochemical and Western blot analyses were performed on excised tumor tissues to determine whether the effects on tumor growth were associated with altered proliferative or apoptotic activity. DhL treatment was associated with a marked decrease in Ki-67 expression relative to the control group, indicating a decrease in tumor cell proliferation (Figure 5C). In contrast, DhL-treated tumors exhibited a pronounced increase in cleaved caspase-3 expression, confirming the activation of apoptosis *in vivo* (see Figures 5D and 5E).

Collectively, these results demonstrate that DhL suppresses OSCC tumor growth *in vivo* and is associated with increased apoptotic signaling, consistent with the pro-apoptotic effects observed *in vitro*. These findings demonstrate the *in vivo* antitumor activity of DhL and support its further evaluation as a potential therapeutic agent for oral squamous cell carcinoma.

## Discussion

OSCC remains a major clinical challenge, with patient outcomes limited by therapeutic resistance and high recurrence rates. Despite progress in multimodal treatment, the five-year survival rate has remained largely unchanged, emphasizing the need for more effective therapeutic strategies. Various natural compounds, such as sesquiterpene lactones, have emerged as promising anticancer candidates with diverse biological activities. Dehydroleucodine (DhL) is a sesquiterpene lactone isolated from *Asteraceae* species, presenting anticancer activity across multiple malignancies through induced apoptosis, cell cycle disruption, and the modulation of oxidative stress 16-20. However, its anticancer effects and underlying mechanism(s) in OSCC remain unclear. This study was the first comprehensive investigation of DhL anticancer effects against OSCC. Our findings indicate that DhL inhibits OSCC cell viability and clonogenicity in association with ROS-related mitochondrial apoptosis and G2/M cell cycle arrest. In an OSCC xenograft model, DhL significantly suppressed tumor growth and was associated with increased apoptotic signaling. Taken together, these findings clarify the mechanisms underlying the anticancer effects of DhL in OSCC.

One key finding in this study was the significant contribution of DhL-induced oxidative stress to apoptosis in OSCC cells. There is a growing body of evidence indicating that the anticancer effects of many sesquiterpene lactones (e.g., parthenolide, costunolide, and artemisinin derivatives) disrupt cellular redox homeostasis and trigger mitochondria-dependent apoptosis 26-29. Consistent with these observations, we demonstrated that DhL treatment elevated intracellular ROS levels, disrupted mitochondrial membrane potential, and activated caspase-3-mediated apoptosis in both HSC3 and SCC4 cells. Importantly, pretreatment with the antioxidant NAC partially rescued cell viability, attenuated apoptosis, and reduced G2/M arrest, suggesting that ROS accumulation is an important contributing factor rather than merely a downstream consequence of DhL-induced cellular stress. This ROS-associated mechanism is consistent with previous reports describing the actions of sesquiterpene lactones in other cancer types 26-29. Interestingly, the apoptotic mechanism in OSCC differs from that reported in Burkitt lymphoma cells, where DhL-induced oxidative stress triggers ferroptotic rather than apoptotic cell death 17. This discrepancy suggests that the mode of DhL-induced cell death may be context-dependent, influenced by baseline redox status, antioxidant capacity, and the expression of cell death regulators. Further studies are warranted to elucidate the determinants of cell death modality in response to DhL across cancer types.

In addition to inducing apoptosis, DhL was shown to cause cell cycle arrest at the G2/M phase, accompanied by downregulation of key mitotic regulators, cyclin B1 and CDK1. This finding is consistent with previous reports demonstrating that DhL induces cell cycle arrest in melanoma and astrocytoma models 18, 19. Moreover, DhL-induced G1/S or G2/M cell cycle arrest was associated with the activation of DNA damage response (DDR) pathways, including the phosphorylation of ATM and H2AX, stabilization of p53 or p73, and upregulation of the cyclin-dependent kinase inhibitor p21 18-20. In the current study, we did not examine these DDR pathways directly; however, it is likely that DhL engages similar mechanisms in OSCC cells. Future investigations should evaluate whether DhL induces DNA damage or activates checkpoint signaling in OSCC, and whether these responses contribute to G2/M arrest and subsequent apoptosis. Note that prolonged cell cycle arrest can sensitize cells to mitochondrial apoptosis by disrupting cellular homeostasis and amplifying stress signals 30. Therefore, it is possible that G2/M arrest and ROS-associated mitochondrial dysfunction cooperatively contribute to the cytotoxic effects of DhL in OSCC cells.

One important observation in this study was the fact that DhL exhibited lower cytotoxic effects on normal human gingival fibroblasts (HGF-1) than on OSCC cells under the tested conditions. At concentrations that significantly reduced OSCC cell viability and clonogenicity, HGF-1 cells retained normal morphology, with no signs of cytotoxicity. This differential response may be related to the elevated baseline oxidative stress commonly observed in cancer cells, which increases susceptibility to further ROS elevation and redox imbalance 31, 32. In contrast, normal cells typically possess robust antioxidant defense systems and lower baseline ROS levels, which improves their ability to tolerate exogenous oxidative stress. Similar selective toxicity has been reported for other sesquiterpene lactones, wherein cancer cells undergo ROS-mediated death while normal cells remain largely unaffected 17, 33-35. Our findings suggest that DhL is more cytotoxic to OSCC cells than to HGF-1 cells under the tested *in vitro* conditions; however, this will have to be confirmed in a broader panel of normal cell models. Although these results support further investigation of DhL, they also underscore the need for additional studies to define its safety profile and translational potential.

The xenograft experiments in this study support our *in vitro* findings, wherein the systemic administration of DhL significantly inhibited OSCC tumor growth *in vivo*. The stable body weight of DhL-treated mice throughout the study indicates high tolerance for the treatment regimen under the tested conditions. Consistent with the observed tumor suppression, immunohistochemical and Western blot analyses of tumor tissues revealed a decrease in Ki-67 expression, indicating reduced proliferative activity and increased cleaved caspase-3 expression, suggesting the activation of apoptosis *in vivo*. Taken together, these findings provide proof-of-concept evidence that DhL suppresses OSCC tumor growth *in vivo* and is associated with increased apoptotic activity. Nevertheless, our *in vivo* findings are insufficient to firmly establish the ROS-related mechanism observed in cultured OSCC cells. Additional studies will be required to define the mechanism underlying DhL activity in animal models.

Despite these promising findings, this work was subject to several limitations that should be considered in the interpretation of our findings. First, the fact that tumor measurements were not performed in a blinded manner may have introduced observer bias. Second, our exclusive use of male mice may have introduced sex-related differences in the antitumor response to DhL. Finally, clinical development will require further preclinical and translational investigations, including extended treatment schedules, dose optimization, formulation and delivery strategies, pharmacokinetic and bioavailability analyses, and comprehensive toxicity profiling in animal models. Combination studies evaluating DhL in conjunction with conventional chemotherapeutic agents or targeted therapies may also help to identify synergistic strategies to improve therapeutic efficacy.

Several important questions pertaining to DhL activity remain unanswered. First, we determined that NAC partially rescues DhL-induced loss of viability, apoptosis, and G2/M arrest, but were unable to determine whether ROS acts as an upstream initiating event or as one of several interacting stress signals involved in DhL-induced cytotoxicity. Second, we did not investigate whether DhL-induced G2/M arrest is mediated through DNA damage response (DDR) pathways or p53/p21-dependent mechanisms, as reported for other cancer types 18-20. Third, DhL demonstrated lower cytotoxicity toward HGF-1 cells than toward OSCC cells; however, a broader panel of normal cell types will be required to define its selectivity and safety profile. Fourth, additional cell death pathways, such as ferroptosis, necroptosis, and autophagy, may also contribute to the anticancer activity of DhL, warranting systematic investigation.

Taken together, this study provides the first comprehensive characterization of the anticancer activity of DhL in OSCC with an account of the molecular events associated with its cytotoxic effects. DhL reduced OSCC cell viability in association with ROS-related mitochondrial apoptotic signaling and G2/M cell cycle arrest with low cytotoxicity toward HGF-1 cells under the tested conditions. These findings support further preclinical investigation of DhL as a potential therapeutic candidate for oral cancer.

## Conclusion

This study provides compelling early evidence that the natural compound dehydroleucodine (DhL) exerts anticancer activity against OSCC. DhL was shown to reduce OSCC cell viability through multiple molecular events, including oxidative stress, disruption of mitochondrial membrane potential, activation of caspase-dependent apoptotic signaling, and G2/M cell cycle arrest. Importantly, DhL exhibited lower cytotoxicity toward normal human gingival fibroblasts than toward OSCC cells. In an OSCC xenograft model, DhL was linked to suppressed tumor growth and increased apoptotic signaling, without a significant loss of body weight under the tested conditions. These findings support further preclinical investigation of DhL as a potential therapeutic candidate for OSCC. Given the growing interest in redox-modulating natural compounds, this study provides mechanistic insights into the anticancer effects of DhL in OSCC as a basis for further investigation in preclinical models.

## Data Availability

The datasets generated and/or analyzed during the current study are available from the corresponding author upon reasonable request. Requests may be directed to Ju-Fang Liu at jufangliu@tmu.edu.tw.
